# Longitudinal Changes in Psychological States in Online Health Community Members: Understanding the Long-Term Effects of Participating in an Online Depression Community

**DOI:** 10.2196/jmir.6826

**Published:** 2017-03-20

**Authors:** Albert Park, Mike Conway

**Affiliations:** ^1^ Department of Biomedical Informatics School of Medicine University of Utah Salt Lake City, UT United States

**Keywords:** mental health, depression, consumer health information, informatics, information science, social support, psychosocial support system, community networks, self-help groups, communications media

## Abstract

**Background:**

Major depression is a serious challenge at both the individual and population levels. Although online health communities have shown the potential to reduce the symptoms of depression, *emotional contagion theory* suggests that negative emotion can spread within a community, and prolonged interactions with other depressed individuals has potential to worsen the symptoms of depression.

**Objective:**

The goals of our study were to investigate longitudinal changes in psychological states that are manifested through linguistic changes in depression community members who are interacting with other depressed individuals.

**Methods:**

We examined emotion-related language usages using the Linguistic Inquiry and Word Count (LIWC) program for each member of a depression community from Reddit. To measure the changes, we applied linear least-squares regression to the LIWC scores against the interaction sequence for each member. We measured the differences in linguistic changes against three online health communities focusing on positive emotion, diabetes, and irritable bowel syndrome.

**Results:**

On average, members of an online depression community showed improvement in 9 of 10 prespecified linguistic dimensions: “positive emotion,” “negative emotion,” “anxiety,” “anger,” “sadness,” “first person singular,” “negation,” “swear words,” and “death.” Moreover, these members improved either significantly or at least as much as members of other online health communities.

**Conclusions:**

We provide new insights into the impact of prolonged participation in an online depression community and highlight the positive emotion change in members. The findings of this study should be interpreted with caution, because participating in an online depression community is not the sole factor for improvement or worsening of depressive symptoms. Still, the consistent statistical results including comparative analyses with different communities could indicate that the emotion-related language usage of depression community members are improving either significantly or at least as much as members of other online communities. On the basis of these findings, we contribute practical suggestions for designing online depression communities to enhance psychosocial benefit gains for members. We consider these results to be an important step toward a better understanding of the impact of prolonged participation in an online depression community, in addition to providing insights into the long-term psychosocial well-being of members.

## Introduction

Social media, including online health communities, has become a popular platform for individuals to exchange social support and connect with others [1]. Research on the benefits of online health communities highlights psychosocial benefits—such as reduced depression [2,3], anxiety [3,4], stress [3], and negative mood [5]—as well as the development of a higher level of coping [6] and patient empowerment [2,6,7]. However, participants in these studies suffered from mental disorders (eg, depression, anxiety) as secondary symptoms and were contributing to health-related forums focusing on, for example, cancer. Although psychosocial benefits of online health communities have been widely studied, it is unclear whether the members of mental health communities would receive similar benefits. Thus, we studied the longitudinal changes in psychological states in members of a mental health community.

Online mental health communities can be a useful medium for individuals suffering from mental disorders [8], due to social stigma and discrimination surrounding such disorders [9-11]. However, in contrast to the substantial work focusing on the benefits of online health communities, *emotional contagion theory* [12] suggests that prolonged interactions with depressed individuals and their negative emotion can worsen the symptoms of depression. *Emotional contagion theory* is a type of emotional influence that describes the spread of one person’s emotion to other people during social encounters.

Scientific evidence in support of *emotional contagion theory* in online health communities can be illustrated in two concise assertions. First, individuals’ inner emotions have been shown to manifest in their choice of words in writing [13], including textual conversation within online health communities [14]. Second, both negative [15] and positive emotions [16] have been shown to spread even through text-based computer-mediated communication (eg, email, chat) as well as through a textual alert system in social media [17].

In the aforementioned study of spreading positive or negative emotion via a textual alert system in social media [17], findings suggest that individuals’ emotions had spread throughout their social community. Moreover, both short-lived moods evinced in response to outside stimuli and long-term emotional states (ie, general emotional state) of community members can affect the emotions of the community. For example, in an empirical study, members of mental health communities were shown to have significant increases in anxiety, anger, and negative emotions following reports of several celebrity suicides [18]; and both positive and negative long-term emotional states slowly spread across socially connected individuals in a social network [19]. Due to this effect, researchers have suggested that interaction with other depressed individuals is a potential risk factor for depression [20,21].

Negative sentiment has been shown to be highly prevalent and more common than positive sentiment in mental health communities, including a depression community [22]. Thus, members of depression communities are likely to be exposed to other members’ negative emotions. However, as members of online health communities, they are also likely to receive emotional support (ie, positive emotion) [23,24]. Thus, the extent to which one of the effects (ie, spreading of negative emotion or positive emotional support) has a greater impact on long-term participants in an online depression community is of interest. Moreover, understanding the impact of long-term participation in online mental health communities can suggest practical implications related to using, managing, designing, and researching online mental health communities that can improve the experience of online mental health community members who seek essential social support during times of difficulty. Despite the importance in clinical, practical, and public policy implications for mental health, to our knowledge, the investigation on the change of emotional states via linguistic change in depression community members has not been the focus of previous research on online mental health communities.

We aim to fill this gap in the literature with this study and answer two research questions (RQ):

RQ1. How does prolonged participation in an online depression community affect the emotional states of its members?

RQ2. How does this change in emotional states compare with other online health communities? As a control, we selected online health communities focusing on (1) positive emotion, (2) health conditions recognized for their needs for both informational and emotional support, and (3) chronic but non–life-threatening health issues that are not often related to mental disorders.

## Methods

### Community Platform

The data for this study consist of posts (ie, a submission that starts a conversation) and associated comments (ie, a submission that replies to posts or other comments) from several topical focused subreddits (ie, subcommunities). All subreddits found in this study are hosted on the popular social media platform, Reddit. Reddit is a social media platform that had 83 billion page views from more than 88,000 active subcommunities (subreddits) in 2015. Reddit content is accessible on the open Web. This can be contrasted with services like Facebook or specifically health-focused online community like PatientsLikeMe, where data are typically not available on the open Web, but requires user registration to access content. Members of Reddit made more than 73 million individual posts with over 725 million associated comments in the same year [25]. Reddit is highly active and the community allows posters to create submissions using throwaway and unidentifiable accounts. These features are suitable for stigmatized conditions and their discussions that may not be appropriate for identifiable accounts. Due to these two reasons, we examined posts and comments from Reddit for this study.

### Subreddit Selection

The community r/depression, to our knowledge, is the largest and most active depression focused subreddit. It has been active for 7 years with 145,821 subscribers [26] in September 2016. Thus, we selected r/depression as the main community of interest for investigating the effects of prolonged participation in an online depression community to the emotional states of members (RQ1).

To understand the significance of the change of emotional states of r/depression members, we compared r/depression against 3 other subreddits (RQ2). We first selected r/happy, a subreddit focused on sharing happy thoughts and stories. r/happy was created to focus on happy thoughts and, to our knowledge, is the largest and most active positive emotion–focused subreddit that has been active for 8 years with 94,076 subscribers [27] in September 2016. Members who are participating in the positive emotion–focused subreddit should encounter less negative emotion than the r/depression members. Thus, such a community can provide an insight into the impact of prolonged participation in an online health community without much exposure to negative emotions.

Much research on online health communities focused on two types of social support emotional and informational support [24,28-30]. Both support types are important aspects of online health communities. For instance, symptom management is an important aspect for patients with diabetes. Thus, sharing informational support, such as practical information to control one’s diabetic symptoms [29] is a common practice in online diabetes communities. Also in online diabetes communities, sharing emotional support such as motivation and accountability [31] is a common practice to help members continuously manage their illnesses. To better understand the changes of emotional states in r/depression, we compared against r/diabetes, a community with abundant informational and emotional support. To our knowledge, r/diabetes is the largest and most active diabetes community on Reddit. The subreddit has been active for 8 years with 15,623 subscribers in September 2016 [32].

To better understand the effects of prolonged participation in online depression communities, we wanted to compare the depression community against online health communities that are not directly related to mental disorders in order to ensure that the changes of emotional states do not result from the secondary symptoms (ie, mental illnesses) that are associated with the primary illness. However, a confounding factor is that serious illnesses are frequently accompanied with mental illnesses, due to the distress of living with—or being diagnosed with—a serious condition. Although it may be impossible to select an online health community without any exchange of emotional support, one study investigating an online Irritable Bowel Syndrome (IBS) community characterized informational support including symptom interpretation and illness management as the main type of social support exchanged in the community [33]. Thus, we selected r/ibs, a subreddit focused on IBS, as a community focused on a chronic but non–life-threatening health issue that is not often related to mental disorders. In September 2016, r/ibs has been active for 6 years with 5251 subscribers [34] and is the largest and most active IBS community in Reddit, to our knowledge.

### Data

First, we use a dataset [35] released by a Reddit member who collected the data from October 2007 to May 2015. The dataset has been used in a previous study [22]. Second, we extracted posts and associated comments from r/depression, r/happy, r/diabetes, and r/ibs. Third, we removed deleted posts or comments that are labeled as “[deleted]” from the dataset. The contents of posts or comments were deleted by members or the communities, thus we excluded them in our analyses. Fourth, to meet our study aims, we restricted our analysis to members (ie, unique user IDs) with 4 or more submissions (ie, posts and comments). In different studies [28,36], this threshold was used to determine lurkers, who are not yet a regularly contributing member of a community, thus we used the same threshold to determine regular members. Table 1 shows the overall characteristics of datasets that we examined.

**Table 1:** Characteristics of the analyzed datasets from r/depression, r/happy, r/diabetes, and r/ibs

Community	r/depression	r/happy	r/diabetes	r/ibs
Date of data	Dec 2008-May 2015	Jan 2008-May 2015	Jan 2009-May 2015	Aug 2012-May 2015
No. of Submissions	571,864	95,329	167,917	15,978
No. of Members	36,746	8130	4539	923

The work described in this paper was granted an exemption from review by the University of Utah’s Institutional Review Board (IRB), ethics committee, [IRB 00076188] under Exemption 2 as defined in United States Federal Regulations 45 CFR 46.101(b).

### Linguistic Analysis

To understand the change of emotional states, we examined emotion-related language using the Linguistic Inquiry and Word Count (LIWC) program [37]. LIWC analyzes text on a word-by-word basis, counts the predefined linguistic dimension words and then returns each linguistic dimension score as a proportion of the total number of words under analysis. The validity of LIWC’s performance has been established in a large number of studies, including studies measuring perceived positive and negative emotions in online mental health communities [18], online health communities [30], text-based online communication [15,16], and social media [17,38].

LIWC consists of 4 major dimensions: “psychological processes,” “linguistic processes,” “personal concerns,” and “spoken categories.” The most notable linguistic dimension related to emotion is “psychological processes,” which includes “positive emotion” and “negative emotion” as subdimensions that are hosted in “affective processes.” “Negative emotion” has 3 subdimensions: “anxiety,” “anger,” and “sadness.” Both “positive emotion” and “negative emotion” dimensions have been used in a series of studies to measure perceived positive and negative emotion in text-based communication [15-18,30,38,39], which Reddit uses to facilitate communication.

From “linguistic processes,” “negation,” and “swear words” have been used to predict depression in social media [38,39]. “Negation” has also been used to measure agreeableness in text-based online communication [15,16]. One of the most widely used dimensions for predicting depression is the “first person singular” [15,16,18,30,38,39]. The frequent use of “first person singular” indicates a heightened sense of self and is known to correlate with depression [40].

The subdimension, “death” from “personal concerns” has been used to measure suicidal thoughts and discussion in Reddit [18] and “assent” from “spoken categories” was used to measure agreeableness in text-based online communication [15,16] and to predict depression [39]. We made these linguistic dimension selections before the linguistic analysis without any information on the linguistic dimension frequency counts. LIWC was applied to individual posts or comments for each subreddit. A summary of the linguistic dimensions and dimensions’ example words for the current study is shown in Table 2.

**Table 2:** Summary of Linguistic Inquiry and Word Count (LIWC) linguistic dimensions used for this study and example vocabulary.

Linguistic dimensions		Example vocabulary
**Psychological processes**		
	Positive emotion	happy, love, sweet
	Negative emotion	agony, hurt, nasty
	Anxiety	afraid, fearful, nervous
	Anger	abuse, annoyed, hate
	Sadness	crying, grief, sad
**Linguistic processes**		
	First person singular	I, me, mine
	Negation	no, not, never
	Swear words	ass, damn, fuck
**Personal concerns**		
	Death	death, die, kill
**Spoken categories**		
	Assent	agree, okay, yes

### RQ1: Change of Emotional States Analysis and RQ2: Community Comparison Analysis

**Figure 1:**
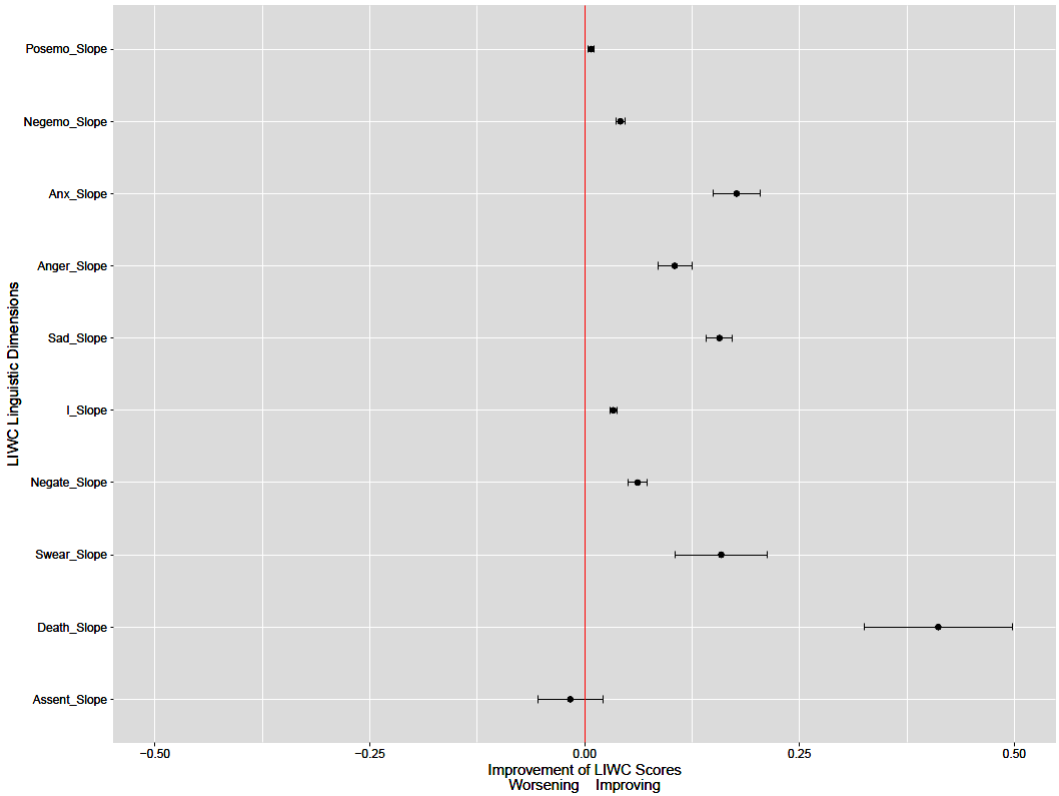
An overview of improvement of each linguistic dimension for r/depression members.

**Figure 2:**
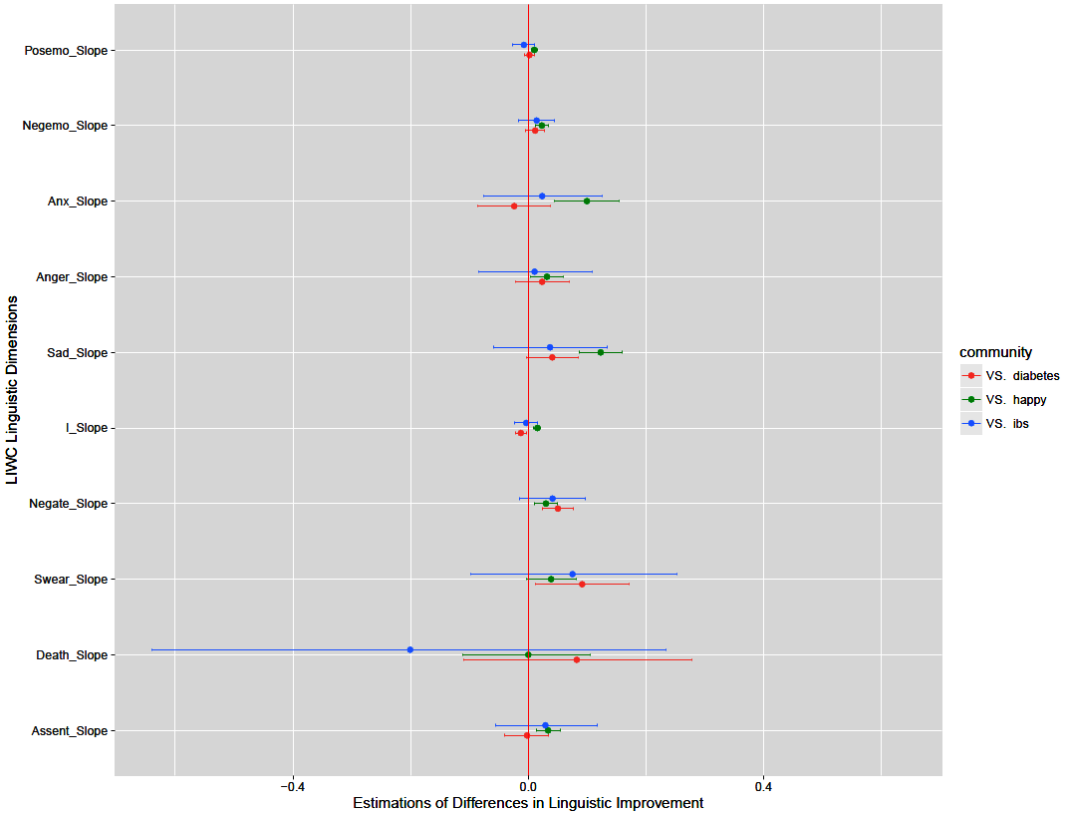
An overview of difference in improvement compared with r/depression.

To measure the change of emotional states of r/depression members (RQ1), we first calculated the scores of prespecified LIWC dimensions. Then, we organized each LIWC score according to the posting time per-member basis for the community. To measure the changes of LIWC scores, we applied linear least-squares regression to the LIWC scores against the interaction sequence (ie, determined by the time of posting) for each member. We elected to perform linear the least-squares regression against the interaction sequence rather than time, because we were interested in the change caused by each interaction rather than time. For each dimension, we calculated mean, median, and 95% confidence interval (CI) of the community to reflect the overall linguistic changes in r/depression members. Then, we converted linguistic change to indicate improvement. Decrease of negative dimensions (ie, “negative emotion,” “anxiety,” “anger,” “sadness,” “first person singular,” “negation,” “swear words,” “death”) indicates improvement, although decrease of positive dimensions (ie, “positive emotion,” “assent”) indicates worsening. To be consistent with figures (Figures 1 and 2), we show improvement for each dimension. Then, we present an overview of improvement for each linguistic dimension along with the statistical significance against the null hypothesis (ie, prolonged participation in an online depression community has no effect on the emotional states of members).

To compare linguistic changes in communities (RQ2), we first carried out the procedure for measuring the change of emotional states for the other three communities. Then, we applied Mann-Whitney *U* tests [41] with the prespecified false discovery rate procedure [42] to *P* values. The comparison of 10 linguistic dimensions raises the need to control for Type I errors (ie, false positives); thus, we applied the false discovery rate procedure. Given the large sample of members, we followed suggestions of a previous study [43] and reported 95% CI and effect sizes (r) for the community comparisons using rank-biserial correlation [44]. We applied nonparametric tests because our data were not normally distributed.

## Results

### RQ 1. How Does Prolonged Participation in an Online Depression Community Affect the Emotional States of its Members?

We captured the average changes of emotional states that are manifest in linguistic changes for r/depression members. Table 3 summarizes the linguistic changes in r/depression members. On average, r/depression members showed improvement in all linguistic dimensions with the exception of “assent.”

**Table 3:** Linguistic changes in r/depression members. Variables are reported as the mean, median, and 95% CIs of word usage change of each dimension for all members.

Linguistic dimensions		r/depression mean, median (CI) n=36,746
**Psychological processes**		
	Positive emotion	0.01, 0.01 (0.004 to 0.01)
	Negative emotion	-0.04, -0.03 (-0.05 to -0.04)
	Anxiety	-0.18, -0.11 (-0.20 to -0.15)
	Anger	-0.10, -0.06 (-0.12 to -0.09)
	Sadness	-0.16, -0.13 (-0.17 to -0.14)
**Linguistic processes**		
	First person singular	-0.03, -0.03 (-0.04 to -0.03)
	Negation	-0.06, -0.05 (-0.07 to -0.05)
	Swear words	-0.16, 0.09 (-0.20 to -0.12)
**Personal concerns**		
	Death	-0.41, -0.21 (-0.47 to -0.35)
**Spoken categories**		
	Assent	-0.02, 0.05 (-0.05 to 0.02)

Then, we converted linguistic changes to indicate improvement and showed an overview of improvement of each linguistic dimension (Figure 1). On the x-axis, less than 0 indicates worsening (ie, decreasing use of positive words or increasing use of negative words), whereas more than 0 indicates an improvement (ie, increasing use of positive words or decreasing use of negative words). The dot indicates the mean of improvement values and the range shows 95% CI. If the range includes 0.0 (eg, “assent”), it suggests that the null hypothesis of prolonged participation in an online depression community has no effect on the emotional states of members cannot be rejected. Our analyses suggested that with the exception of “assent” dimension, members of r/depression showed significant improvement in linguistic changes. No significant change was found in the “assent” dimension.

### RQ 2. How Does This Change in Emotional States Compare With Other Online Communities?

To compare the linguistic changes evident in r/depression to r/happy, r/diabetes, and r/ibs, we first calculated the mean, median, and CIs of word usage change for each linguistic dimension for each subreddit (Table 4).

**Table 4:** Linguistic changes in members from r/happy, r/ibs, and r/diabetes. Variables are reported as the mean, median, and 95% CIs of word usage change of each dimension for all members.

Linguistic dimensions		r/happy mean, median (CI) n=8130	r/diabetes mean, median (CI) n=4539	r/ibs mean, median (CI) n=923
**Psychological processes**				
	Positive emotion	0.005, 0.004 (0.0005-0.01)	0.01, 0.01 (-0.01 to 0.02)	0.02, 0.02 (-0.005 to 0.05)
	Negative emotion	-0.01, -0.01 (-0.03 to 0.001)	-0.02, -0.02 (-0.04 to 0.003)	-0.005, -0.02 (-0.05 to 0.03)
	Anxiety	0.002, -0.003 (-0.06 to 0.06)	-0.26, -0.15 (-0.35 to -0.17)	-0.18, -0.08 (-0.31 to -0.04)
	Anger	-0.02, -0.02 (-0.06 to 0.02)	-0.11, -0.04 (-0.18 to -0.03)	-0.13, -0.06 (-0.27 to 0.01)
	Sadness	0.01, -0.003 (-0.04 to 0.07)	-0.18, -0.10 (-0.25 to -0.10)	-0.15, -0.11 (-0.33 to 0.04)
**Linguistic processes**				
	First person singular	-0.01, -0.01 (-0.02 to -0.01)	-0.09, -0.04 (-0.11 to -0.07)	-0.03, -0.03 (-0.06 to -0.008)
	Negation	-0.02, -0.01 (-0.04 to 0.01)	0.02, 0.002 (-0.02 to 0.05)	-0.008, -0.01 (-0.08 to 0.06)
	Swear words	-0.03, -0.02 (-0.09 to 0.03)	-0.01, 0.005 (-0.20 to 0.18)	-0.06, -0.01 (0.44 to 0.33)
**Personal concerns**				
	Death	-0.52, -0.17 (-0.69 to -0.36)	-0.93, -0.22 (-1.35 to -0.51)	-0.98, -0.56 (-1.73 to -0.23)
**Spoken categories**				
	Assent	0.02, 0.01 (-0.01 to 0.05)	-0.03, 0.05 (-0.10 to 0.05)	0.04, 0.02 (-0.09 to 0.18)

On average, members of these 3 subreddits showed significant improvement in linguistic changes with increasing number of interactions. Four exceptions are “anxiety” and “sadness” from r/happy and “negation” and “assent” from r/diabetes. Then, we compared the 3 subreddits to r/depression (Table 5).

**Table 5:** Community comparison of average linguistic changes against r/depression.

Linguistic dimensions		versus r/happy	versus r/diabetes	versus r/ibs
**Psychological processes**				
	Positive emotion	r/depression U^a^=151428562 *P*=3.9e-05 *P*_adj_^b^=9.8e-05 CI^c^=0.005-0.015 *r*=0.04	r/depression U^a^ =83095368.5 *P*=.63 *P*_adj_^b^ =.70 CI^c^ =-0.01 to 0.01 *r*=0.09	r/ibs U^a^ =16588110 *P*=.43 *P*_adj_^b^ =.70 CI^c^ =-0.03 to 0.01 *r*=0.02
	Negative emotion	r/depression U^a^ =118117817 *P*=2.7e-05 *P*_adj_^b^ =8.9 e-05 CI^c^ =-0.03 to -0.01 *r*=0.21	r/depression U^a^ =77894854 *P*=.16 *P*_adj_^b^ =.31 CI^c^ =-0.03 to 0.004 *r*=0.14	r/depression U^a^ =16023844.5 *P*=.37 *P*_adj_^b^ =.70 CI^c^ =-0.05 to 0.02 *r*=0.05
	Anxiety	r/depression U^a^ =42155753 *P*=3.1e-04 *P*_adj_^b^ =6.2e-04 CI^c^ =-0.15 to -0.05 *r*=0.72	r/depression U^a^ = 49336254 *P*=.44 *P*_adj_^b^ =.55 CI^c^ =-0.04 to 0.09 *r*=0.47	r/depression U^a^ =11317589.5 *P*=.62 *P*_adj_^b^ =.78 CI^c^ =-0.13 to 0.08 *r*=0.33
	Anger	r/depression U^a^ =72410492.5 *P*=.02 *P*_adj_^b^ =.03 CI^c^ =-0.06 to -0.004 *r*=0.52	r/depression U^a^ =54287645.5 *P*=.30 *P*_adj_^b^ =.50 CI^c^ =-0.07 to 0.02 *r*=0.40	r/depression U^a^ =11059329.5 *P*=.82 *P*_adj_^b^ =.82 CI^c^ =-0.11 to 0.08 *r*=0.35
	Sadness	r/depression U^a^ =54288775 *P*=1.8e-11 *P*_adj_^b^ =1.8e-10 CI^c^ =-0.16 to -0.09 *r*=0.64	r/depression U^a^ =59564322.5 *P*=.07 *P*_adj_^b^ =.17 CI^c^ =-0.08 to 0.002 *r*=0.34	r/depression U^a^ =11862037.5 *P*=.45 *P*_adj_^b^ =.70 CI^c^ =-0.13 to 0.06 *r*=0.3
**Linguistic processes**				
	First person singular	r/depression U^a^ =137694516.5 *P*=5.6e-07 *P*_adj_^b^ =2.8e-06 CI^c^ =-0.02 to -0.009 *r*=0.08	r/diabetes^d^ U^a^ =84591685.5 *P*=.01 *P*_adj_^b^ =.03 CI^c^ =0.004 to 0.02 *r*=0.11	r/ibs U^a^ =16945136 *P*=.72 *P*_adj_^b^ =.80 CI^c^ =-0.02 to 0.02 *r*=0.01
	Negation	r/depression U^a^ =96382143.5 *P*=.003 *P*_adj_^b^ =4.7e-03 CI^c^ =-0.05 to 0.01 *r*=0.35	r/depression^e^ U^a^ =71632553 *P*=.0002 *P*_adj_^b^ =.002 CI^c^ =-0.08 to -0.02 *r*=0.21	r/depression U^a^ =14855011 *P*=.14 *P*_adj_^b^ =.70 CI^c^ =-0.10 to 0.01 *r*=0.12
	Swear words	r/depression U^a^ =40086953 *P*=.08 *P*_adj_^b^ =.09 CI^c^ =-0.08 to 0.003 *r*=0.73	r/depression U^a^ =30918210.5 *P*=.02 *P*_adj_^b^ =.07 CI^c^ =-0.17 to -0.01 *r*=0.66	r/ibs U^a^ =5505668 *P*=.40 *P*_adj_^b^ =.70 CI^c^ =-0.25 to 0.10 *r*=0.67
**Personal concerns**				
	Death	r/happy U^a^ =14379783 *P*=.95 *P*_adj_^b^ =.95 CI^c^ =-0.11 to 0.11 *r*=0.9	r/depression U^a^ =15714429 *P*=.40 *P*_adj_^b^ =.55 CI^c^ =-0.28 to 0.11 *r*=0.83	r/ibs U^a^ =2476626 *P*=.37 *P*_adj_^b^ =.70 CI^c^ =-0.23 to 0.64 *r*=0.86
**Spoken categories**				
	Assent	r/depression U^a^ =70286713.5 *P*=.001 *P*_adj_^b^ =.002 CI^c^ =0.01-0.05 *r*=0.56	r/depression U^a^ =45944192 *P*=.89 *P*_adj_^b^ =.89 CI^c^ = -0.04 to 0.03 *r*=0.49	r/depression U^a^ =8873209.5 *P*=.49 *P*_adj_^b^ =.70 CI^c^ =-0.05 to 0.12 *r*=0.49

^a^U: Mann-Whitney *U* test values.

^b^*P*_adj_: adjusted *P* values.

^c^95% CI values.

Subreddit comparisons indicate that members of r/depression improved either significantly or at least as much as (ie, no significant difference) members of other online health communities. In “positive emotion,” “negative emotion,” “anxiety,” “anger,” “sadness,” “first person singular,” “negation,” and “assent,” r/depression showed significantly more improvement than r/happy. “Swear words” and “death” showed no significant difference.

The difference in improvements between r/depression and r/diabetes were not significant, with 2 exceptions. The subreddit r/diabetes improved significantly as compared with r/depression in “first person singular,” although r/depression improved significantly as compared with r/diabetes in “negation.” There were no significant differences between r/ depression and r/ibs for all dimensions.

At this point, we converted linguistic changes to indicate improvement compared with r/depression and showed an overview of the differences with respect to each linguistic dimension (Figure 2). The dot indicates the sample estimate and the range shows 95% CI. If the range includes 0.0, it indicates that the difference between the means is not significant.

## Discussion

### Principal Findings on Change of Emotional States for Members of an Online Depression Community

This study has been deeply informed by recent literature focusing on the psychosocial benefits of online health communities [1-7]. We have also utilized *emotional contagion theory* [12] and empirical studies on *emotional contagion theory* [15-17,19,20] to develop this study. We address gaps in the previous literature by shedding light on the question of whether members of an online depression community experience positive or negative emotional changes after prolonged participation.

The linguistic changes have been shown to occur when emotional states are shifting [38]. In the online depression community we analyzed, we found that members of the depression community generally showed an increased use of positive words and decreased use of negative words as the number of interactions increased. Despite prolonged interactions with other depressed individuals, r/depression members’ emotional states were found to have become more positive. For r/depression members, on average all linguistic dimensions were improving with the exception of “assent.” The change in usage of “assent” was found to be insignificant; and as “assent” belongs to “spoken categories,” we believe many vocabularies in “assent” were used in a communicative sense, such as answering questions, a typical type of communication in online health communities [45].

Consistent with previous studies [1-7], we found a similar trend—more use of positive emotion words and less use of negative emotion words—in 3 other Reddit communities. These Reddit communities are different in size (ie, number of members), length of active years, and level of activity, while focusing on different types of health issues. Moreover, we chose these different communities on the basis of the literature that suggested a different composition of social support in these communities. Yet, in all 4 communities, we found members generally used more positive emotion words and less negative emotion words as the number of interactions increased.

The comparison of communities indicates that members of r/depression improved either significantly or at least as much as (ie, no significant difference) members of other online health communities. One unusual finding was the lack of improvement in r/happy, especially when compared with other online health communities. We believe because r/happy members were encouraged to share only positive thoughts and stories from the start, they had limited capacity for positive linguistic changes. However, further investigation is needed for a better understanding of this phenomenon.

### Practical Implications for Online Community Use, Research, and Design

Many online communities utilize the efforts of moderators and even community members to monitor their communities. However, detecting changes of emotional states in members can be difficult with such manual efforts, given that the knowledge of members’ (1) previous emotional states and (2) style of writing are required, as well as (3) linguistic changes can be subtle. An adaptation of our linguistic analysis method can be a basis for detecting subtle linguistic changes in individuals in massive scale networks.

Moderators of online health communities have mentally demanding tasks [46]. An automated detection of linguistic changes could reduce the mental burden of moderators and improve the quality of moderation. For example, such a system could alert moderators and members of any undesirable linguistic changes and allow them to provide timely support. Furthermore, a similar system can assist individual members and the whole community. For individual members, a similar system could be used to raise self-awareness of their changes of linguistic or emotional state. As for communities, a similar system could alert when a community-wide emotional shift occurs. Alerting of a negative shift can allow timely intervention for the whole community, whereas alerting of a positive shift provides opportunities for researchers and community managers to study the positive change. As studies on *emotional contagion theory* [15-17,19,20] demonstrated, mental health communities carry a potential for a community-wide spread of positive and negative emotions.

### User Privacy

Although Reddit data (like Twitter) are public, and research using public social media is typically granted exemption from review by IRBs in the US context, ethical considerations—particularly with respect to privacy—remain [47-49]. In this paper, in order to protect user anonymity, we did not present user identifiable information (eg, direct quotations, usernames).

### Limitations and Future Directions

One of the limitations in our study is that we had a dataset with 4 communities within the Reddit platform. Although Reddit is a widely used platform, it is more frequently used by young males [50,51]. Therefore, results may not be generalizable to other communities. Also, selection bias of our subjects exists. Members who choose to participate in r/depression are not necessarily representative of the depression population. Similarly, we do not have any evidence that members of r/depression are clinically diagnosed with depression. Thus, we cannot conclude that participating in an online depression community is beneficial for all depression patients. However, r/depression is worth studying given the size of r/depression, the prevalence of depressive disorder and the increasing popularity of online mental health communities.

Although beyond the scope of this study, an investigation with respect to changes of emotion in users of different systems such as Internet-based interventions for depression [52] or text-based crisis counselor system [53] is important for individuals suffering from depression. It is unlikely that any of these avenues could reach the entire population of depressed individuals [9]; thus, understanding the extent to which emotional change occurs within demographic segment in each of these different avenues is important.

Another limitation is the performance of LIWC in measuring emotional states. Although LIWC has been validated and used in many studies [15-18,30,38-40] including a study using Reddit data [18], it is important to note LIWC’s limitations, such as missing potentially important misspellings and slang, which are common in online communities [54]. The findings of this study should be interpreted with caution, especially when leveraging the findings in the clinical context. Furthermore, participation in an online depression community is not the sole factor for improvement or worsening of depressive symptoms. It is also unclear why members stay or leave in this particular online depression community and how their engagement affects the results. Although a previous study suggested that emotional support (ie, positive emotion) helps members stay with an online health community [30], we cannot conclude that participating in online health communities directly caused an improvement in emotional states of members without directly asking the participants. However, this is a general limitation of any secondary analyses. Still, the consistent statistical results could indicate that depressive symptoms of members of an online depression community were improving. Future work could bolster the current study’s findings by using mixed methods such as surveys and interviews and ask the members of online depression communities about the changes in their depressive symptoms and measure depression using standardized methods like the patient health questionnaire or primary care evaluation of mental disorders [55].

We considered word usages as independent observations; nevertheless, correlations could exist in the word usages among members depending on the topic of conversation. For instance, in a conversation about death, members who are in the conversation are more likely to use words related to death regardless of their emotional states. Conversational topics in online communities, however, are known to change [56]. Thus, examining the effects of emotional states in changing topics and their associated sentiments could support our findings. Moreover, individuals could participate in multiple subreddits and use multiple user IDs, both of which violate our independent variable assumption. We also acknowledge that our large sample size could have inflated the statistical significance levels. Thus, we reported 95% CIs and effect sizes as well as compared with other online health communities to aid the interpretation of results.

Finally, our dataset did not capture passive activities like lurking. Although it has been suggested that lurkers experience fewer benefits than active participants [3], it is unclear if lurkers have less emotional changes and how their changes of emotion can affect the community when they become active participants. Understanding emotional changes in lurkers could deepen our understanding in future work.

### Conclusions

We provide new insights into the impact of prolonged participation in an online depression community and highlight the positive emotion change in members. Our findings suggest that 3 other Reddit communities focusing on positive emotion, diabetes, and IBS also have a similar positive emotion change in members. The comparison of communities indicates that the emotion-related language usage of depression community members improved either significantly or at least as much as (ie, no significant difference) members of other online communities. On the basis of these findings, we contribute practical suggestions for designing online depression communities to enhance psychosocial gains for members. We consider these results to be an important step toward developing a better understanding of the impact of prolonged participation in an online depression community on emotional health, in addition to providing insights into the long-term psychosocial well-being of members.

## Abbreviations

IBS: irritable bowel syndrome
LIWC: Linguistic Inquiry and Word Count
CI: Confidence Interval
